# Diagnostic Sign of Choleraic Diarrhœa

**Published:** 1855-01

**Authors:** 


Dyagnostic sign of Choleraic Diarrhœa.—The New York Academy of Medicine calls attention to the fact, that the diarrhœa of epidemic Cholera is always *painless*, while that of common Cholera is more or less painful.—*Nashville Journal.*

